# A mixed methods approach to assess the reliability of a test for the success of blinding in a surgical randomised controlled trial (RCT)

**DOI:** 10.1186/1745-6215-12-S1-A71

**Published:** 2011-12-13

**Authors:** Caroline E Boulind, Kerry Avery, Chris Metcalfe, Nader K Francis, Jane M Blazeby

**Affiliations:** 1 University of Bristol, School of Social & Community Medicine, Canynge Hall, 39 Whatley Road, Bristol, BS8 2PS, UK

## Background

Blinding participants in randomised controlled trials (RCTs) with patient reported outcomes is important to reduce bias and widely used. The success of blinding is rarely tested, but may be assessed with tools such as the Bang Blinding Index (BBI). There is controversy, however, about the value and interpretation of test results.

## Objectives

To investigate the reliability of the BBI in a feasibility RCT comparing two types of pain relief following colorectal cancer surgery.

## Methods

Participants were asked to guess which trial arm they were in within a day of surgery and at discharge. Responses could include arm A, arm B or ‘don’t know’ (DK). Participants responding DK were also asked to give a ‘forced guess’ and reasons for responses were documented. Data were analysed using the Bang Blinding Index (BBI). Proportions of correct guesses for the two arms of the trial were compared using Fisher’s exact test. Two analyses were performed, first using the original responses (including DK), then replacing DK with the forced guesses.

Audio-recorded semi-structured interviews with participants were undertaken after discharge, exploring the reasons behind participants’ guesses and beliefs about treatment allocation. The interviews were analysed using a constant comparison method. Results from the qualitative analysis were triangulated with the BBI data.

## Results

Twenty six participants were included. In arm A (n=13), 62% correctly guessed treatment allocation, compared to 70% in arm B (n=13, p=0.41). The BBI result for people in arm A was 0.15: 15% more correct guesses than expected by chance. In arm B the result was 0.31, suggesting that 31% more than expected by chance correctly guessed treatment allocation. When DK responses were replaced with the forced guesses, the proportions of correct guesses changed to 69% for arm A and 63% for arm B (p=0.43). The corresponding BBI results were 0.38 and 0.08 respectively.

Qualitative interviews suggested that there is variable understanding of the reason for blinding among trial participants, but that they were accepting of the need for blinding and that their guesses reflected true beliefs about their treatment allocation.

## Conclusions

The BBI can be used to reliably estimate the rate of unblinding among trial participants and is likely to accurately reflect participant beliefs about treatment allocation; however DK responses may not represent successful blinding, and the use of information from forced guesses is important for the correct interpretation of the BBI.

